# 2601. A 14-year retrospective study of pulmonary nocardiosis in AIDS patients, Northeastern Thailand

**DOI:** 10.1093/ofid/ofad500.2216

**Published:** 2023-11-27

**Authors:** wilawan thipmontree, Yupin Suputtamongkol

**Affiliations:** Maharat Nakhon Ratchasima Hospital, Amphur Muang, Nakhon Ratchasima, Thailand; Mahidol University, Bangkok, Buriram, Thailand

## Abstract

**Background:**

Nocardiosis is an opportunistic infection in immunocompromised individuals, particularly patients with acquired immune deficiency syndrome (AIDS). Clinical manifestations of nocardiosis are wide-ranging but the most common is pneumonia.

**Methods:**

This 14 year retrospective study included *Nocardia* species infected adult patients (≥15 years) with at least 28 days of treatment follow-up. This study aims to compare clinical manifestations and survival analysis of cultured confirmed pulmonary nocardiosis among AIDS and non-AIDS patients.

**Results:**

From 2009 to 2022, 237 patients had culture confirmed *Nocardia* species infection, of which 22 were excluded from this study because 13 had cutaneous nocardiosis and nine had pulmonary nocardiosis with follow-up less than 28 days. A total of 215 confirmed pulmonary nocardiosis were recruited to the analysis, 97(45.1%) were HIV-infected and 118 (54.9%) were non-HIV. All HIV-infected patient has CD4 count below 200 cell/mm^3^ (range 1-198, 1-15%), known as AIDS. Most of them were male with a mean age of 37 (range 15-83) years. One hundred fifty of the 215 (69.7%) patients underwent brain imaging. Sixteen cases (10.7%) had brain abscess. Disseminated nocardiosis (brain abscess, bacteremia, cutaneous abscess) was found in equal proportions in AIDS (15/97, 15.5%) and non-AIDS patients (17/118, 14.4%), while pulmonary nocardiosis with local invasion (empyema thoracis, pericardial effusion) was more common in AIDS (11/97, 11.3%) than non-AIDS patients (9/118, 7.6%). Overall and 28 days mortality were 116 (53.9%) and 88 (40.9%), respectively. In Cox’s proportional hazard analysis, AIDS was the independent factor associated with 28 days mortality in pulmonary nocardiosis (adjusted HR 2.62; 95%CI, 1.38 to 4.98) and those who underwent brain imaging (adjusted HR 2.75; 95%CI, 1.23 to 6.15) after adjusted by sex, age, duration of illness, respiratory failure, shock, multi-lobar pneumonia, disseminated nocardiosis, and combination antibiotic therapy.

**Figure:**
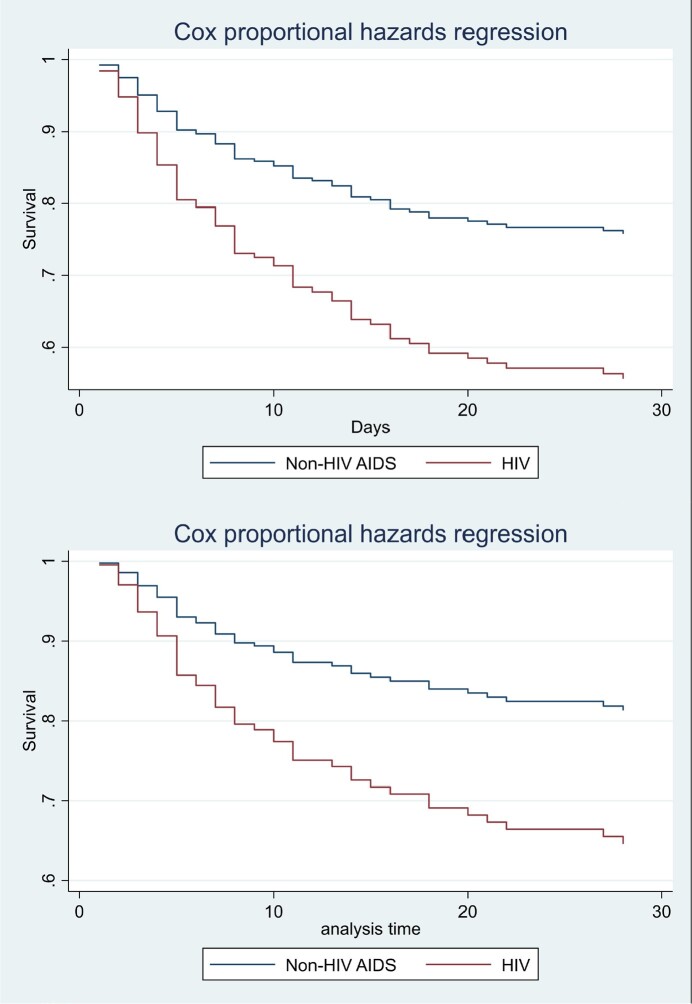

**Conclusion:**

Pulmonary nocardiosis is a serious opportunistic infection in AIDS patient and has shown significantly higher mortality than non-AIDS. Therefore, nocardiosis should always be considered as a cause of pneumonia in AIDS patients in order to prompt aggressive treatment.

**Disclosures:**

**Yupin Suputtamongkol, MD**, Abbott Labs: Grant/Research Support|Abbott Labs: Mahidol University is partner in the Abbott Pandemic Defense Coalition

